# The non-slip suture: tissue approximation in high-tension wounds using a stepwise instrument-based suture method

**DOI:** 10.1308/rcsann.2025.0082

**Published:** 2025-10-01

**Authors:** T Shoaib, J Lucocq

**Affiliations:** ^1^ La Belle Forme Clinic, UK; ^2^ Western General Hospital, UK

## Background

Achieving reliable dermal approximation under tension remains a challenge but is important to optimise wound healing, prevent wound dehiscence and minimise postoperative infection.^1,2^ This paper describes a novel and reproducible instrument-based suturing technique for tissue approximation under tension.

## Technique

1. Approximation

**Figure 1:**
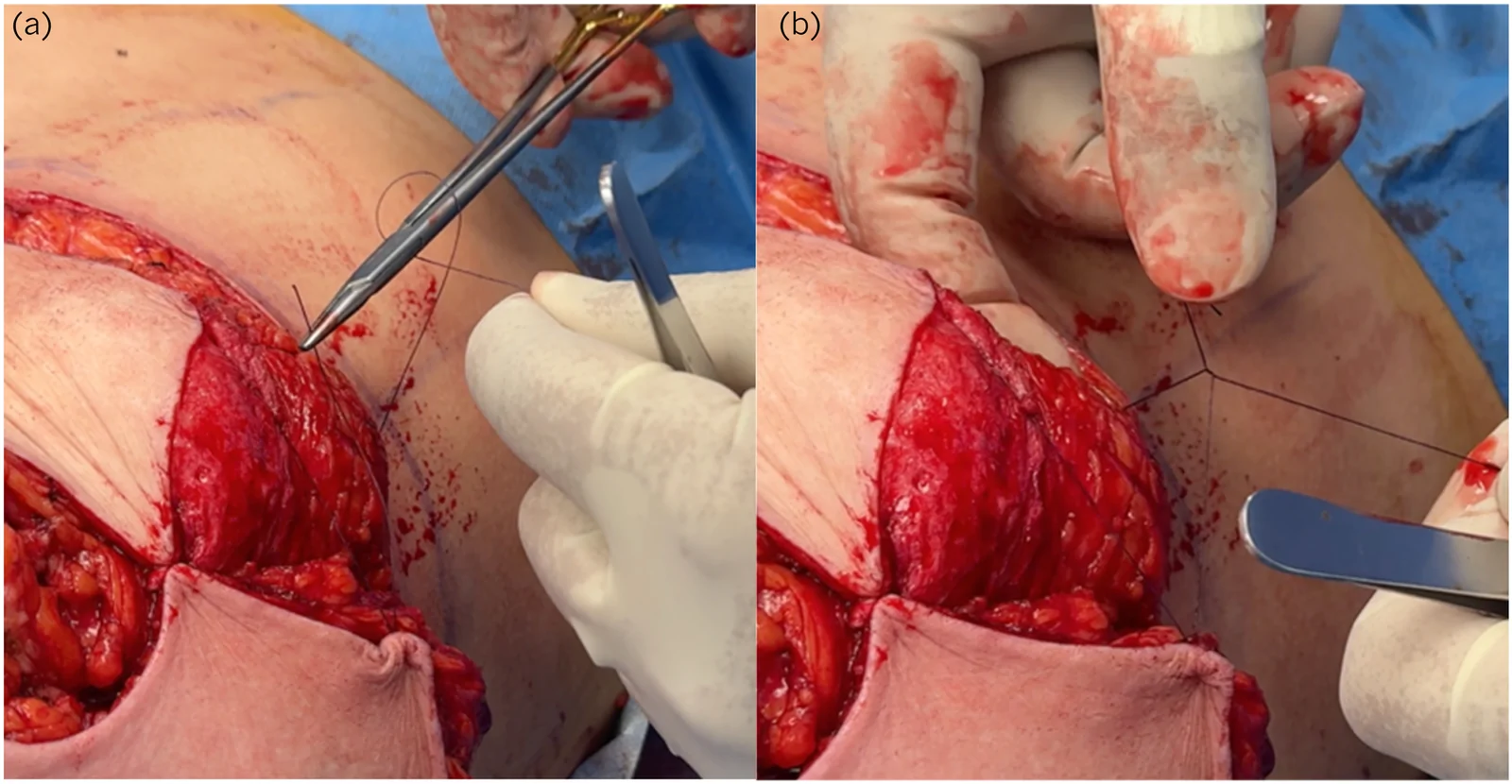
(a,b) The first throw of the suture is made in a breast reduction case.

After the bites of tissue on each side of the wound are taken, optionally, one loop of the surgical knot is tied and tightened with as many throws as the surgeon prefers (Figure 1a,b). To aid approximation, the needle holder can push subcutaneous tissue gently into the wound. The suture material must remain held in position to maintain tissue apposition; otherwise, apposition will fail.

2. Formation of the loop

**Figure 2:**
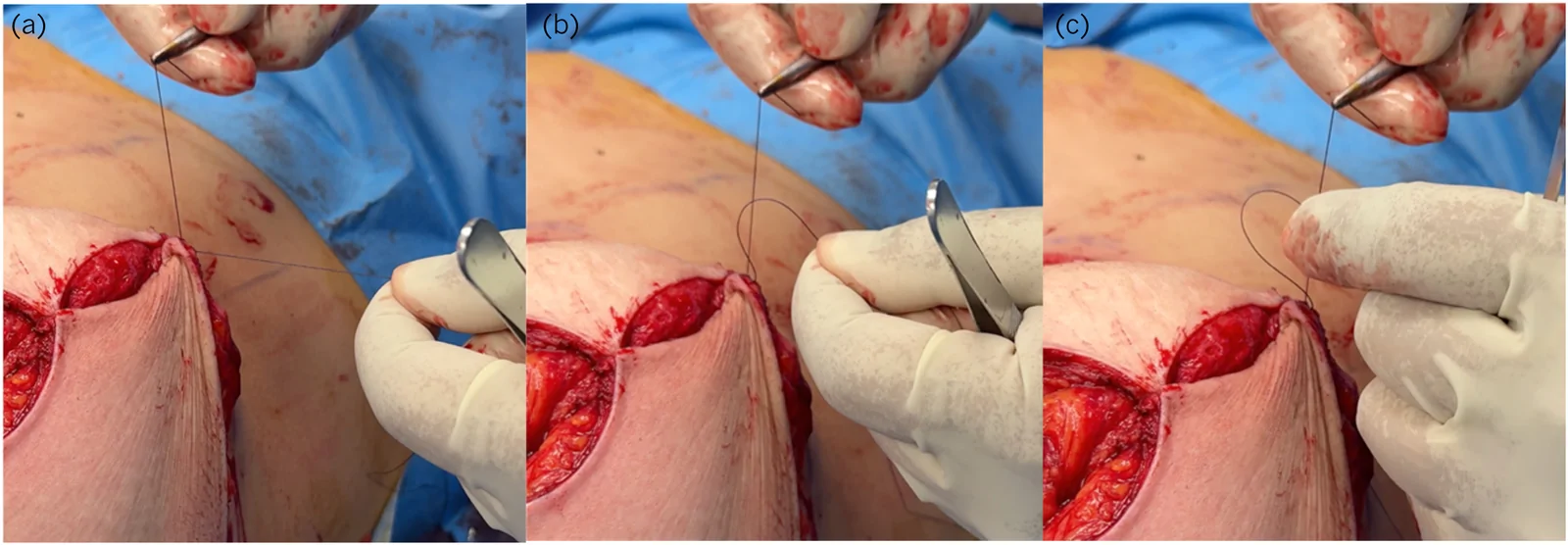
(a–c) Formation of the loop.

The needle-end of the suture is held by the non-dominant hand (Figure 2a). The needle holder passes the free-end to the non-dominant hand and is held approximately 5cm from the very tip. A loop is created between the two suture ends (Figure 2b) and is held between the index finger and thumb (Figure 2c).

3. Formation of the knot

**Figure 3:**
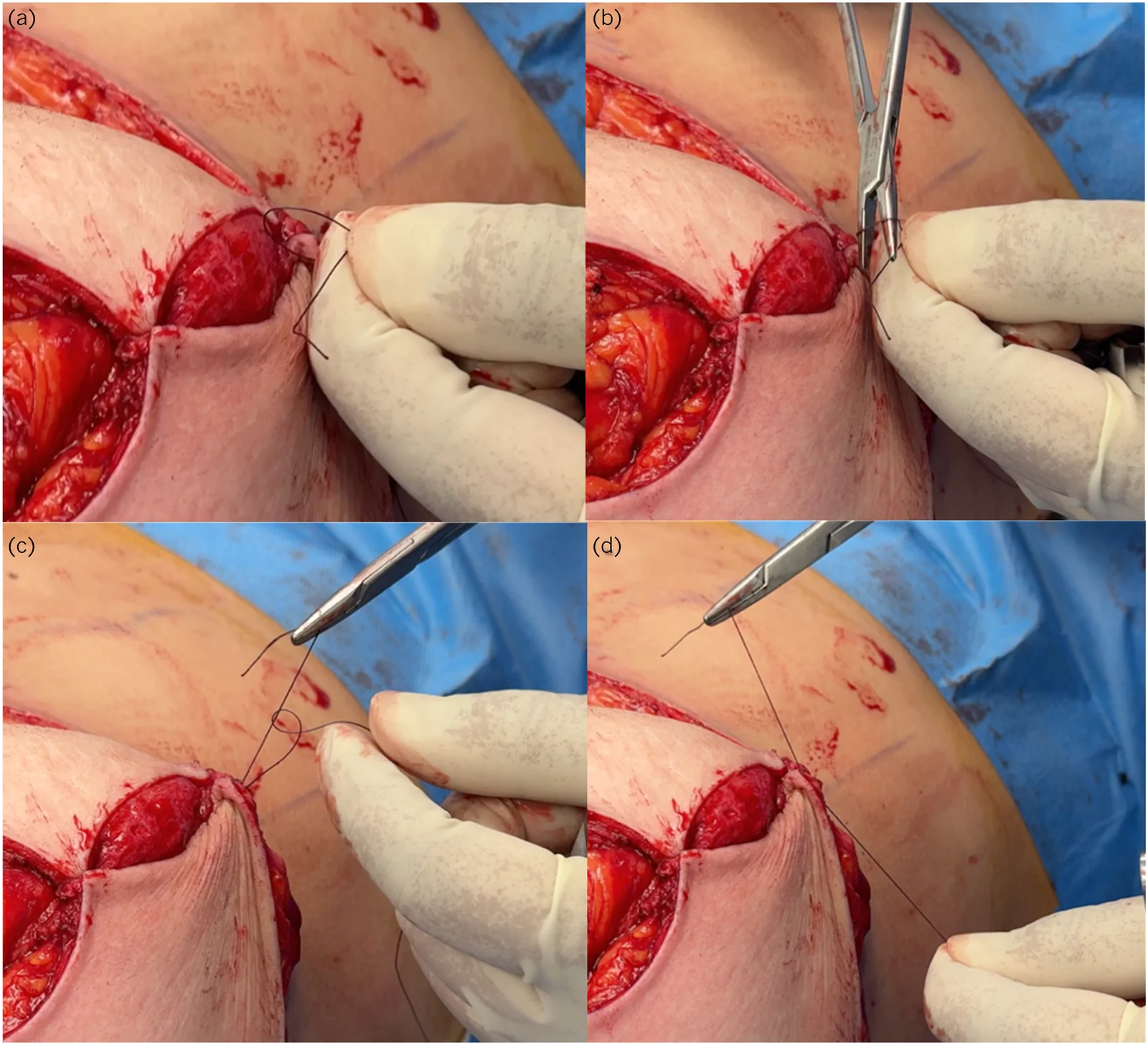
(a–d) The knot is secured.

The non-dominant hand is supinated to rotate the tip of the free-end away from the operator (Figure 3a). The needle holder is then passed through the loop to grasp the tip of the free-end (Figure 3b). The free-end is pulled through the loop and tension is then transferred to the needle holder (Figure 3c). The non-dominant hand can guide the knot into position (Figure 3d). At least two knots are required before moving to an alternative technique for suture knot tying. The technique is equally applicable to left-handed surgeons, who may perform the steps in a mirror-image fashion.

## Discussion

This non-slip instrument-based suturing technique for secure dermal approximation under tension can be applied across a range of procedures (e.g. abdominoplasty, mammoplasty). In comparison with hand-tying and previous instrument-based techniques, this technique is more economical of suture length, maintains absolute control of the spatial position of the free-end of the suture and enables the use of the needle holder to improve tissue manipulation.^3,4^

## References

[1] Gantwerker EA, Hom DB. Skin: histology and physiology of wound healing. *Facial Plast Surg Clin North Am* 2011; **19**: 441–453. 21856533 10.1016/j.fsc.2011.06.009

[2] Singh S, Young A, McNaught CE. The physiology of wound healing. *Surgery (Oxford)* 2017; **35**: 473–477.

[3] Revington P, Bowyer RC. Sutures: the economics of knot tying techniques. *Ann R Coll Surg Engl* 1994; **76**: 281. 7598399

[4] Tillo O, Nawinne M, Oudit D. Approximating tissue under tension using the slipped square sliding knot. *Ann R Coll Surg Engl* 2008; **90**: 258–259. 10.1308/003588408X285720g PMC2430445 18437720

